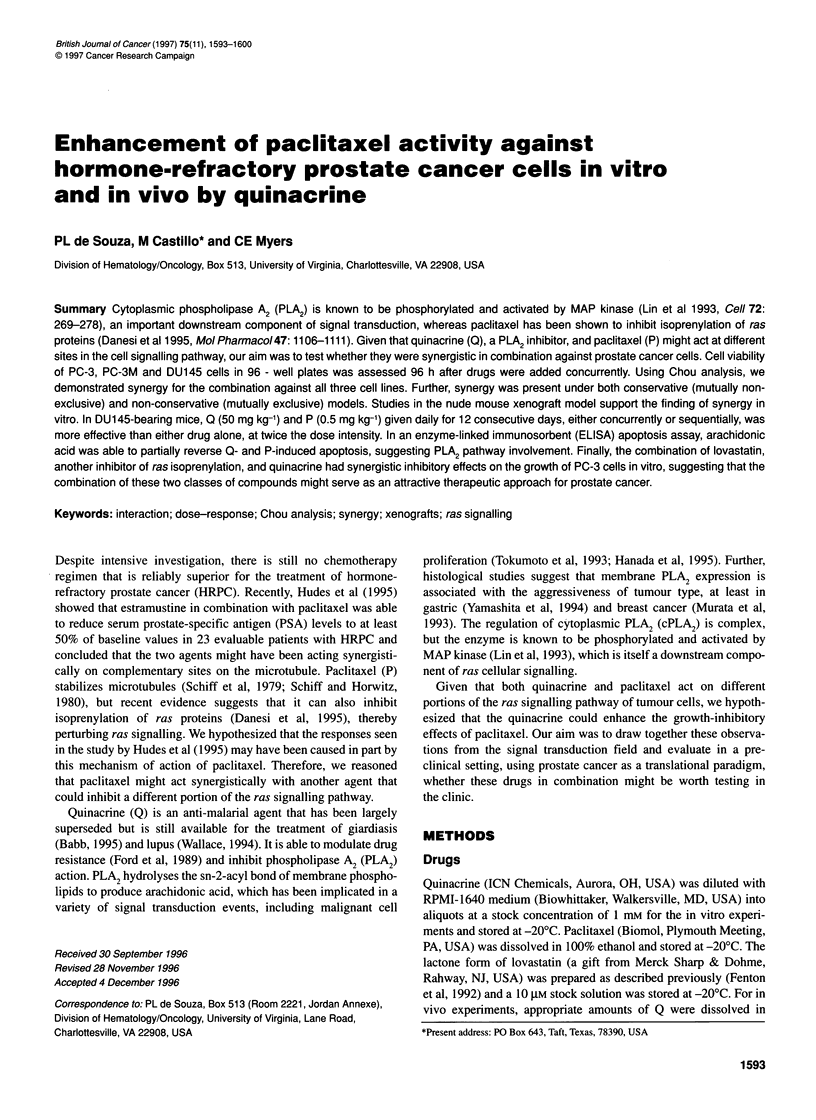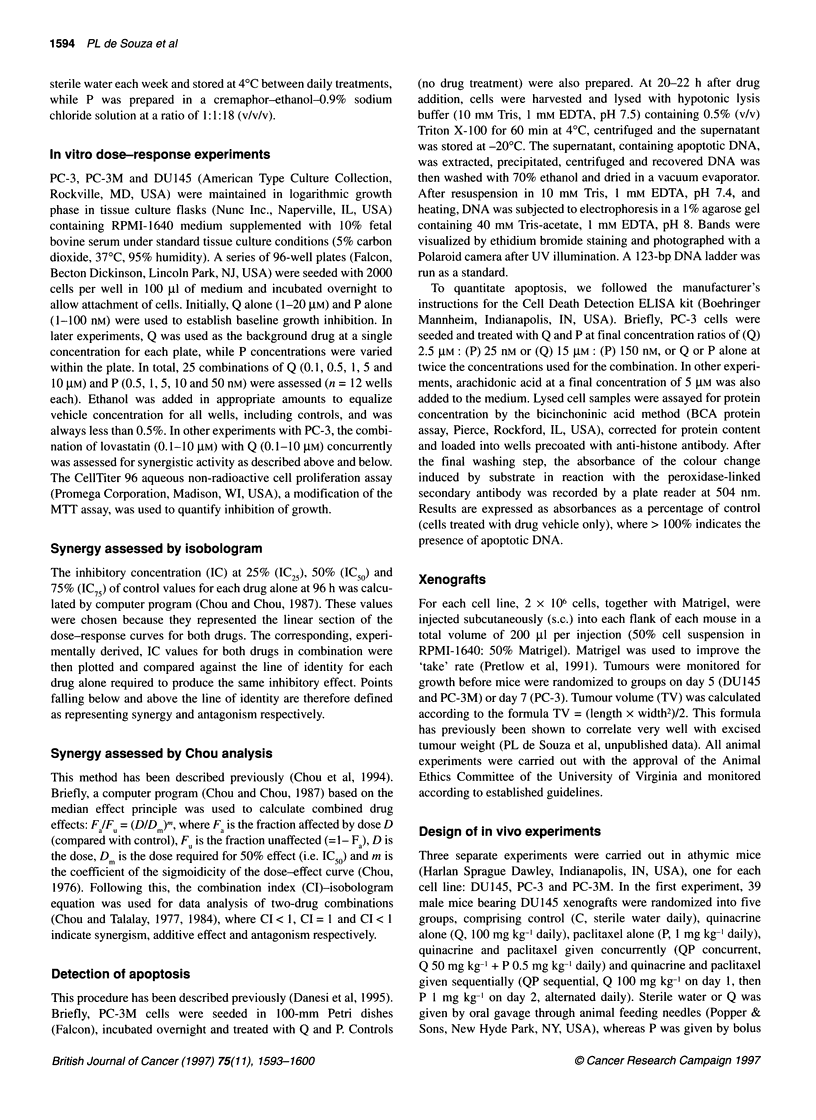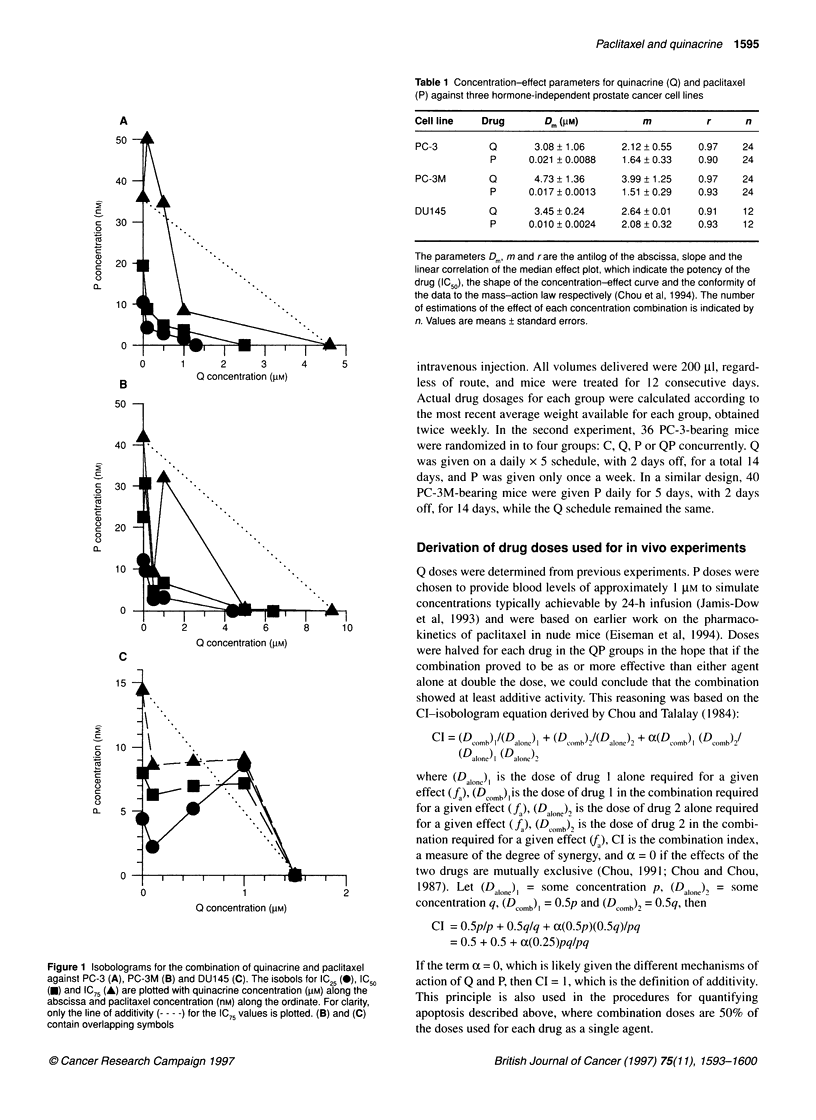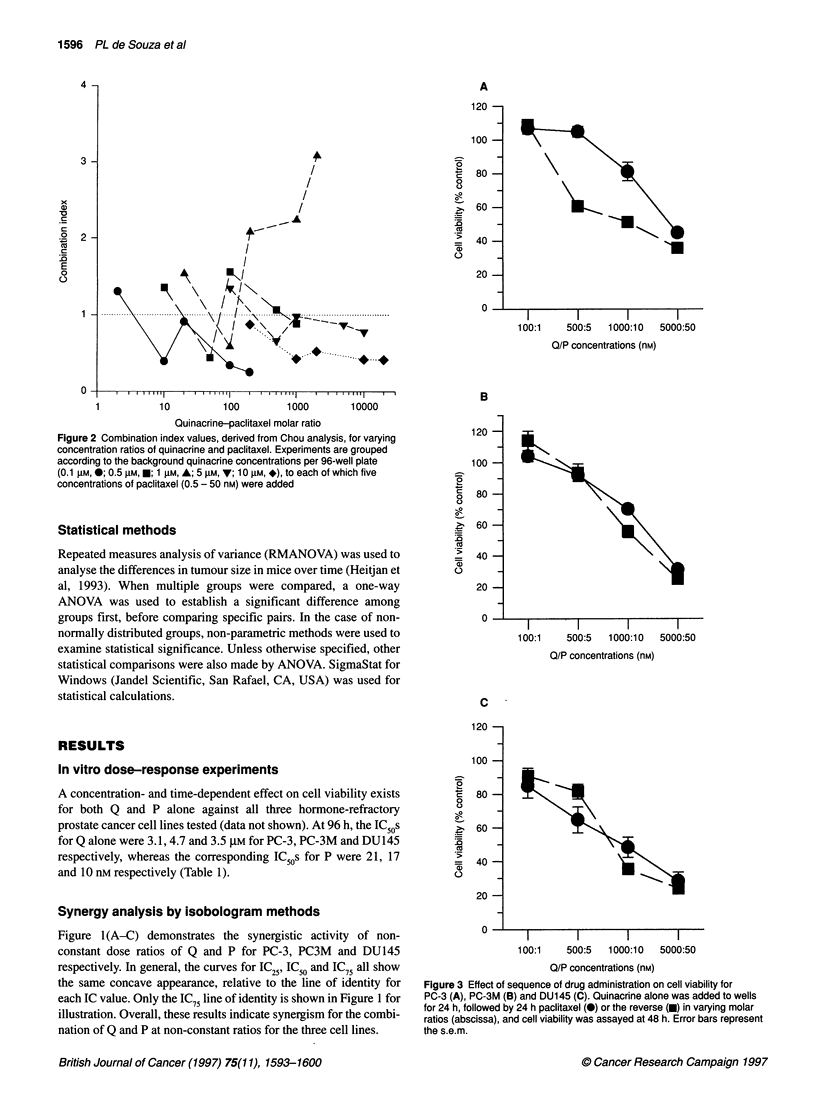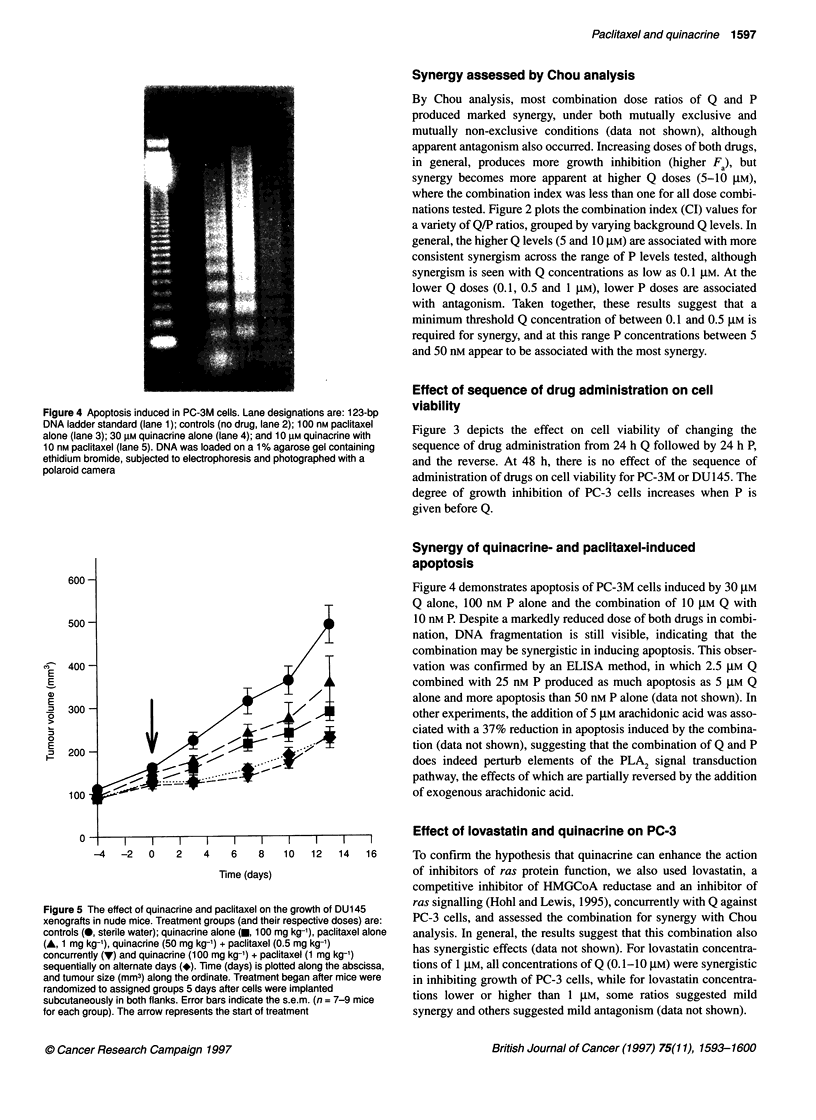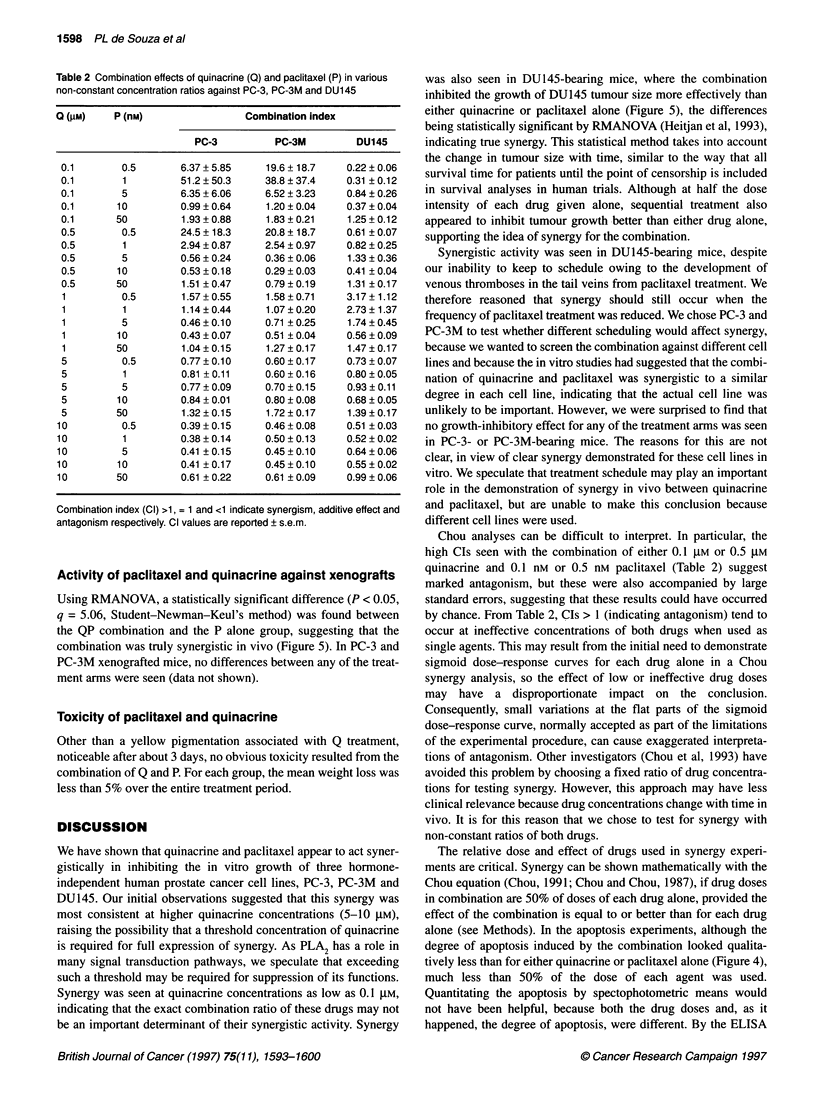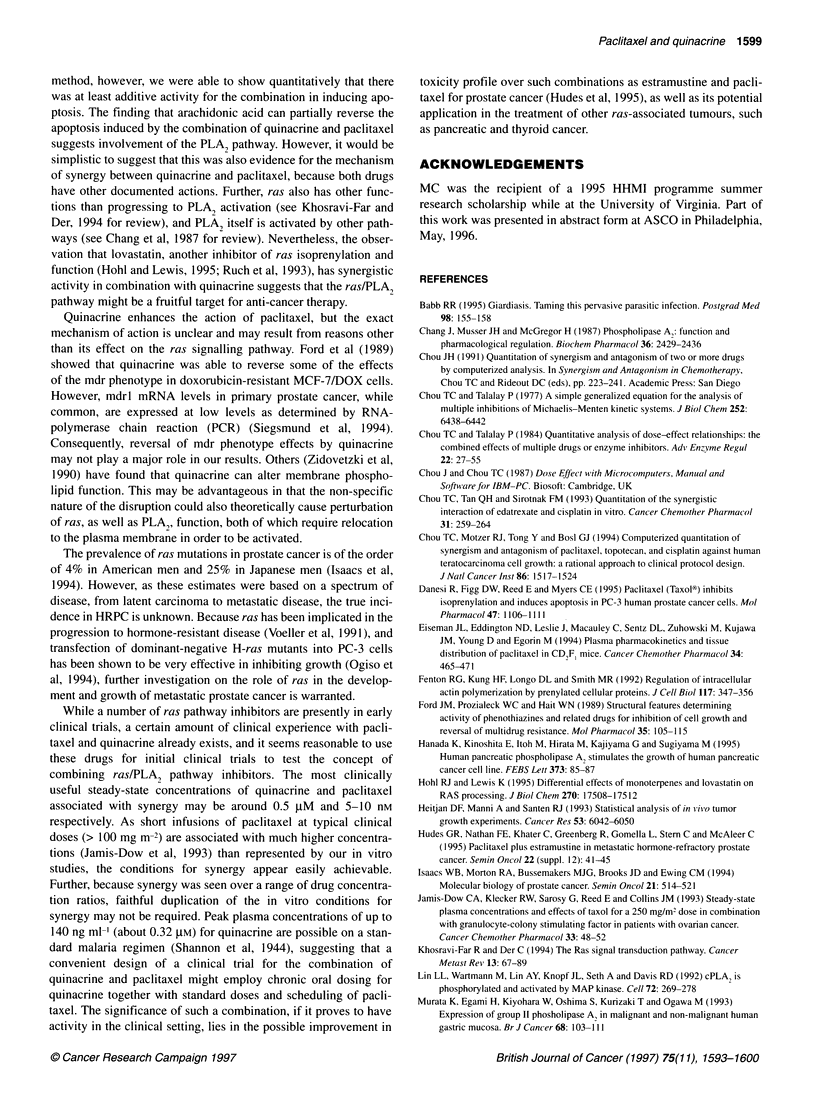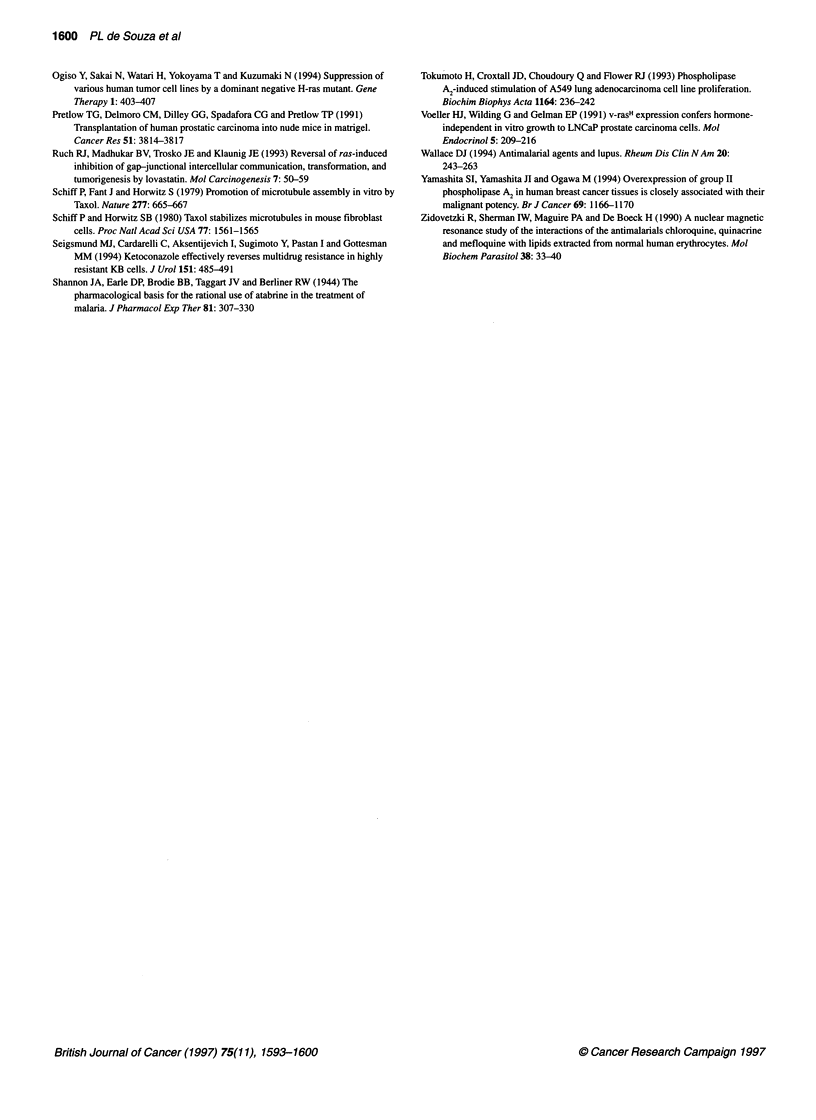# Enhancement of paclitaxel activity against hormone-refractory prostate cancer cells in vitro and in vivo by quinacrine.

**DOI:** 10.1038/bjc.1997.272

**Published:** 1997

**Authors:** P. L. de Souza, M. Castillo, C. E. Myers

**Affiliations:** Division of Hematology/Oncology, University of Virginia, Charlottesville 22908, USA.

## Abstract

**Images:**


British Journal of Cancer (1997) 75(11), 1593–1600
© 1997 Cancer Research Campaign

### Enhancement of paclitaxel activity against hormone-refractory prostate cancer cells in vitro and in vivo by quinacrine

**PL de Souza, M Castillo* and CE Myers**

Division of Hematology/Oncology, Box 513, University of Virginia, Charlottesville, VA 22908, USA

**Summary** Cytoplasmic phospholipase $A_2$ ($PLA_2$) is known to be phosphorylated and activated by MAP kinase (Lin et al 1993, *Cell* **72**: 269–278), an important downstream component of signal transduction, whereas paclitaxel has been shown to inhibit isoprenylation of *ras* proteins (Danesi et al 1995, *Mol Pharmacol* **47**: 1106–1111). Given that quinacrine (Q), a $PLA_2$ inhibitor, and paclitaxel (P) might act at different sites in the cell signalling pathway, our aim was to test whether they were synergistic in combination against prostate cancer cells. Cell viability of PC-3, PC-3M and DU145 cells in 96 - well plates was assessed 96 h after drugs were added concurrently. Using Chou analysis, we demonstrated synergy for the combination against all three cell lines. Further, synergy was present under both conservative (mutually non-exclusive) and non-conservative (mutually exclusive) models. Studies in the nude mouse xenograft model support the finding of synergy in vitro. In DU145-bearing mice, Q (50 mg $kg^{-1}$) and P (0.5 mg $kg^{-1}$) given daily for 12 consecutive days, either concurrently or sequentially, was more effective than either drug alone, at twice the dose intensity. In an enzyme-linked immunosorbent (ELISA) apoptosis assay, arachidonic acid was able to partially reverse Q- and P-induced apoptosis, suggesting $PLA_2$ pathway involvement. Finally, the combination of lovastatin, another inhibitor of *ras* isoprenylation, and quinacrine had synergistic inhibitory effects on the growth of PC-3 cells in vitro, suggesting that the combination of these two classes of compounds might serve as an attractive therapeutic approach for prostate cancer.

**Keywords:** interaction; dose–response; Chou analysis; synergy; xenografts; *ras* signalling

Despite intensive investigation, there is still no chemotherapy regimen that is reliably superior for the treatment of hormone-refractory prostate cancer (HRPC). Recently, Hudes et al (1995) showed that estramustine in combination with paclitaxel was able to reduce serum prostate-specific antigen (PSA) levels to at least 50% of baseline values in 23 evaluable patients with HRPC and concluded that the two agents might have been acting synergistically on complementary sites on the microtubule. Paclitaxel (P) stabilizes microtubules (Schiff et al, 1979; Schiff and Horwitz, 1980), but recent evidence suggests that it can also inhibit isoprenylation of *ras* proteins (Danesi et al, 1995), thereby perturbing *ras* signalling. We hypothesized that the responses seen in the study by Hudes et al (1995) may have been caused in part by this mechanism of action of paclitaxel. Therefore, we reasoned that paclitaxel might act synergistically with another agent that could inhibit a different portion of the *ras* signalling pathway.

Quinacrine (Q) is an anti-malarial agent that has been largely superseded but is still available for the treatment of giardiasis (Babb, 1995) and lupus (Wallace, 1994). It is able to modulate drug resistance (Ford et al, 1989) and inhibit phospholipase $A_2$ ($PLA_2$) action. $PLA_2$ hydrolyses the sn-2-acyl bond of membrane phospholipids to produce arachidonic acid, which has been implicated in a variety of signal transduction events, including malignant cell proliferation (Tokumoto et al, 1993; Hanada et al, 1995). Further, histological studies suggest that membrane $PLA_2$ expression is associated with the aggressiveness of tumour type, at least in gastric (Yamashita et al, 1994) and breast cancer (Murata et al, 1993). The regulation of cytoplasmic $PLA_2$ ($cPLA_2$) is complex, but the enzyme is known to be phosphorylated and activated by MAP kinase (Lin et al, 1993), which is itself a downstream component of *ras* cellular signalling.

Given that both quinacrine and paclitaxel act on different portions of the *ras* signalling pathway of tumour cells, we hypothesized that the quinacrine could enhance the growth-inhibitory effects of paclitaxel. Our aim was to draw together these observations from the signal transduction field and evaluate in a pre-clinical setting, using prostate cancer as a translational paradigm, whether these drugs in combination might be worth testing in the clinic.

### METHODS

### Drugs

Quinacrine (ICN Chemicals, Aurora, OH, USA) was diluted with RPMI-1640 medium (Biowhittaker, Walkersville, MD, USA) into aliquots at a stock concentration of 1 mM for the in vitro experiments and stored at –20°C. Paclitaxel (Biomol, Plymouth Meeting, PA, USA) was dissolved in 100% ethanol and stored at –20°C. The lactone form of lovastatin (a gift from Merck Sharp & Dohme, Rahway, NJ, USA) was prepared as described previously (Fenton et al, 1992) and a 10 μM stock solution was stored at –20°C. For in vivo experiments, appropriate amounts of Q were dissolved in

*Received 30 September 1996*
*Revised 28 November 1996*
*Accepted 4 December 1996*

*Correspondence to:* PL de Souza, Box 513 (Room 2221, Jordan Annexe), Division of Hematology/Oncology, University of Virginia, Lane Road, Charlottesville, VA 22908, USA

*Present address: PO Box 643, Taft, Texas, 78390, USA

sterile water each week and stored at 4°C between daily treatments, while P was prepared in a cremaphor–ethanol–0.9% sodium chloride solution at a ratio of 1:1:18 (v/v/v).

### In vitro dose–response experiments

PC-3, PC-3M and DU145 (American Type Culture Collection, Rockville, MD, USA) were maintained in logarithmic growth phase in tissue culture flasks (Nunc Inc., Naperville, IL, USA) containing RPMI-1640 medium supplemented with 10% fetal bovine serum under standard tissue culture conditions (5% carbon dioxide, 37°C, 95% humidity). A series of 96-well plates (Falcon, Becton Dickinson, Lincoln Park, NJ, USA) were seeded with 2000 cells per well in 100 µl of medium and incubated overnight to allow attachment of cells. Initially, Q alone (1–20 µM) and P alone (1–100 nM) were used to establish baseline growth inhibition. In later experiments, Q was used as the background drug at a single concentration for each plate, while P concentrations were varied within the plate. In total, 25 combinations of Q (0.1, 0.5, 1, 5 and 10 µM) and P (0.5, 1, 5, 10 and 50 nM) were assessed ($n$ = 12 wells each). Ethanol was added in appropriate amounts to equalize vehicle concentration for all wells, including controls, and was always less than 0.5%. In other experiments with PC-3, the combination of lovastatin (0.1–10 µM) with Q (0.1–10 µM) concurrently was assessed for synergistic activity as described above and below. The CellTiter 96 aqueous non-radioactive cell proliferation assay (Promega Corporation, Madison, WI, USA), a modification of the MTT assay, was used to quantify inhibition of growth.

### Synergy assessed by isobologram

The inhibitory concentration (IC) at 25% ($IC_{25}$), 50% ($IC_{50}$) and 75% ($IC_{75}$) of control values for each drug alone at 96 h was calculated by computer program (Chou and Chou, 1987). These values were chosen because they represented the linear section of the dose–response curves for both drugs. The corresponding, experimentally derived, IC values for both drugs in combination were then plotted and compared against the line of identity for each drug alone required to produce the same inhibitory effect. Points falling below and above the line of identity are therefore defined as representing synergy and antagonism respectively.

### Synergy assessed by Chou analysis

This method has been described previously (Chou et al, 1994). Briefly, a computer program (Chou and Chou, 1987) based on the median effect principle was used to calculate combined drug effects: $F_a/F_u = (D/D_m)^m$, where $F_a$ is the fraction affected by dose $D$ (compared with control), $F_u$ is the fraction unaffected (=1– $F_a$), $D$ is the dose, $D_m$ is the dose required for 50% effect (i.e. $IC_{50}$) and $m$ is the coefficient of the sigmoidicity of the dose–effect curve (Chou, 1976). Following this, the combination index (CI)–isobologram equation was used for data analysis of two-drug combinations (Chou and Talalay, 1977, 1984), where CI < 1, CI = 1 and CI < 1 indicate synergism, additive effect and antagonism respectively.

### Detection of apoptosis

This procedure has been described previously (Danesi et al, 1995). Briefly, PC-3M cells were seeded in 100-mm Petri dishes (Falcon), incubated overnight and treated with Q and P. Controls (no drug treatment) were also prepared. At 20–22 h after drug addition, cells were harvested and lysed with hypotonic lysis buffer (10 mM Tris, 1 mM EDTA, pH 7.5) containing 0.5% (v/v) Triton X-100 for 60 min at 4°C, centrifuged and the supernatant was stored at –20°C. The supernatant, containing apoptotic DNA, was extracted, precipitated, centrifuged and recovered DNA was then washed with 70% ethanol and dried in a vacuum evaporator. After resuspension in 10 mM Tris, 1 mM EDTA, pH 7.4, and heating, DNA was subjected to electrophoresis in a 1% agarose gel containing 40 mM Tris-acetate, 1 mM EDTA, pH 8. Bands were visualized by ethidium bromide staining and photographed with a Polaroid camera after UV illumination. A 123-bp DNA ladder was run as a standard.

To quantitate apoptosis, we followed the manufacturer's instructions for the Cell Death Detection ELISA kit (Boehringer Mannheim, Indianapolis, IN, USA). Briefly, PC-3 cells were seeded and treated with Q and P at final concentration ratios of (Q) 2.5 µM : (P) 25 nM or (Q) 15 µM : (P) 150 nM, or Q or P alone at twice the concentrations used for the combination. In other experiments, arachidonic acid at a final concentration of 5 µM was also added to the medium. Lysed cell samples were assayed for protein concentration by the bicinchoninic acid method (BCA protein assay, Pierce, Rockford, IL, USA), corrected for protein content and loaded into wells precoated with anti-histone antibody. After the final washing step, the absorbance of the colour change induced by substrate in reaction with the peroxidase-linked secondary antibody was recorded by a plate reader at 504 nm. Results are expressed as absorbances as a percentage of control (cells treated with drug vehicle only), where > 100% indicates the presence of apoptotic DNA.

### Xenografts

For each cell line, 2 × $10^6$ cells, together with Matrigel, were injected subcutaneously (s.c.) into each flank of each mouse in a total volume of 200 µl per injection (50% cell suspension in RPMI-1640: 50% Matrigel). Matrigel was used to improve the 'take' rate (Pretlow et al, 1991). Tumours were monitored for growth before mice were randomized to groups on day 5 (DU145 and PC-3M) or day 7 (PC-3). Tumour volume (TV) was calculated according to the formula TV = (length × width$^2$)/2. This formula has previously been shown to correlate very well with excised tumour weight (PL de Souza et al, unpublished data). All animal experiments were carried out with the approval of the Animal Ethics Committee of the University of Virginia and monitored according to established guidelines.

### Design of in vivo experiments

Three separate experiments were carried out in athymic mice (Harlan Sprague Dawley, Indianapolis, IN, USA), one for each cell line: DU145, PC-3 and PC-3M. In the first experiment, 39 male mice bearing DU145 xenografts were randomized into five groups, comprising control (C, sterile water daily), quinacrine alone (Q, 100 mg kg$^{-1}$ daily), paclitaxel alone (P, 1 mg kg$^{-1}$ daily), quinacrine and paclitaxel given concurrently (QP concurrent, Q 50 mg kg$^{-1}$ + P 0.5 mg kg$^{-1}$ daily) and quinacrine and paclitaxel given sequentially (QP sequential, Q 100 mg kg$^{-1}$ on day 1, then P 1 mg kg$^{-1}$ on day 2, alternated daily). Sterile water or Q was given by oral gavage through animal feeding needles (Popper & Sons, New Hyde Park, NY, USA), whereas P was given by bolus

© Cancer Research Campaign 1997

**Figure 1** Isobolograms for the combination of quinacrine and paclitaxel against PC-3 (**A**), PC-3M (**B**) and DU145 (**C**). The isobols for $IC_{25}$ (●), $IC_{50}$ (■) and $IC_{75}$ (▲) are plotted with quinacrine concentration (μM) along the abscissa and paclitaxel concentration (nM) along the ordinate. For clarity, only the line of additivity (- - - -) for the $IC_{75}$ values is plotted. (**B**) and (**C**) contain overlapping symbols

**Table 1** Concentration–effect parameters for quinacrine (Q) and paclitaxel (P) against three hormone-independent prostate cancer cell lines

| Cell line | Drug | $D_m$ (μM) | *m* | *r* | *n* |
|---|---|---|---|---|---|
| PC-3 | Q | 3.08 ± 1.06 | 2.12 ± 0.55 | 0.97 | 24 |
| | P | 0.021 ± 0.0088 | 1.64 ± 0.33 | 0.90 | 24 |
| PC-3M | Q | 4.73 ± 1.36 | 3.99 ± 1.25 | 0.97 | 24 |
| | P | 0.017 ± 0.0013 | 1.51 ± 0.29 | 0.93 | 24 |
| DU145 | Q | 3.45 ± 0.24 | 2.64 ± 0.01 | 0.91 | 12 |
| | P | 0.010 ± 0.0024 | 2.08 ± 0.32 | 0.93 | 12 |

The parameters $D_m$, *m* and *r* are the antilog of the abscissa, slope and the linear correlation of the median effect plot, which indicate the potency of the drug ($IC_{50}$), the shape of the concentration–effect curve and the conformity of the data to the mass–action law respectively (Chou et al, 1994). The number of estimations of the effect of each concentration combination is indicated by *n*. Values are means ± standard errors.

intravenous injection. All volumes delivered were 200 μl, regardless of route, and mice were treated for 12 consecutive days. Actual drug dosages for each group were calculated according to the most recent average weight available for each group, obtained twice weekly. In the second experiment, 36 PC-3-bearing mice were randomized in to four groups: C, Q, P or QP concurrently. Q was given on a daily × 5 schedule, with 2 days off, for a total 14 days, and P was given only once a week. In a similar design, 40 PC-3M-bearing mice were given P daily for 5 days, with 2 days off, for 14 days, while the Q schedule remained the same.

### Derivation of drug doses used for in vivo experiments

Q doses were determined from previous experiments. P doses were chosen to provide blood levels of approximately 1 μM to simulate concentrations typically achievable by 24-h infusion (Jamis-Dow et al, 1993) and were based on earlier work on the pharmacokinetics of paclitaxel in nude mice (Eiseman et al, 1994). Doses were halved for each drug in the QP groups in the hope that if the combination proved to be as or more effective than either agent alone at double the dose, we could conclude that the combination showed at least additive activity. This reasoning was based on the CI–isobologram equation derived by Chou and Talalay (1984):

$$CI = (D_{comb})_1/(D_{alone})_1 + (D_{comb})_2/(D_{alone})_2 + \alpha(D_{comb})_1\,(D_{comb})_2/(D_{alone})_1\,(D_{alone})_2$$

where $(D_{alone})_1$ is the dose of drug 1 alone required for a given effect ($f_a$), $(D_{comb})_1$ is the dose of drug 1 in the combination required for a given effect ($f_a$), $(D_{alone})_2$ is the dose of drug 2 alone required for a given effect ($f_a$), $(D_{comb})_2$ is the dose of drug 2 in the combination required for a given effect ($f_a$), CI is the combination index, a measure of the degree of synergy, and $\alpha = 0$ if the effects of the two drugs are mutually exclusive (Chou, 1991; Chou and Chou, 1987). Let $(D_{alone})_1$ = some concentration *p*, $(D_{alone})_2$ = some concentration *q*, $(D_{comb})_1 = 0.5p$ and $(D_{comb})_2 = 0.5q$, then

$$CI = 0.5p/p + 0.5q/q + \alpha(0.5p)(0.5q)/pq$$
$$= 0.5 + 0.5 + \alpha(0.25)pq/pq$$

If the term $\alpha = 0$, which is likely given the different mechanisms of action of Q and P, then CI = 1, which is the definition of additivity. This principle is also used in the procedures for quantifying apoptosis described above, where combination doses are 50% of the doses used for each drug as a single agent.

© Cancer Research Campaign 1997

Figure 2 Combination index values, derived from Chou analysis, for varying concentration ratios of quinacrine and paclitaxel. Experiments are grouped according to the background quinacrine concentrations per 96-well plate (0.1 μM, ●; 0.5 μM, ■; 1 μM, ▲; 5 μM, ▼; 10 μM, ◆), to each of which five concentrations of paclitaxel (0.5 – 50 nM) were added

### Statistical methods

Repeated measures analysis of variance (RMANOVA) was used to analyse the differences in tumour size in mice over time (Heitjan et al, 1993). When multiple groups were compared, a one-way ANOVA was used to establish a significant difference among groups first, before comparing specific pairs. In the case of non-normally distributed groups, non-parametric methods were used to examine statistical significance. Unless otherwise specified, other statistical comparisons were also made by ANOVA. SigmaStat for Windows (Jandel Scientific, San Rafael, CA, USA) was used for statistical calculations.

### RESULTS

### In vitro dose–response experiments

A concentration- and time-dependent effect on cell viability exists for both Q and P alone against all three hormone-refractory prostate cancer cell lines tested (data not shown). At 96 h, the $IC_{50}$s for Q alone were 3.1, 4.7 and 3.5 μM for PC-3, PC-3M and DU145 respectively, whereas the corresponding $IC_{50}$s for P were 21, 17 and 10 nM respectively (Table 1).

### Synergy analysis by isobologram methods

Figure 1(A–C) demonstrates the synergistic activity of non-constant dose ratios of Q and P for PC-3, PC3M and DU145 respectively. In general, the curves for $IC_{25}$, $IC_{50}$ and $IC_{75}$ all show the same concave appearance, relative to the line of identity for each IC value. Only the $IC_{75}$ line of identity is shown in Figure 1 for illustration. Overall, these results indicate synergism for the combination of Q and P at non-constant ratios for the three cell lines.

Figure 3 Effect of sequence of drug administration on cell viability for PC-3 (A), PC-3M (B) and DU145 (C). Quinacrine alone was added to wells for 24 h, followed by 24 h paclitaxel (●) or the reverse (■) in varying molar ratios (abscissa), and cell viability was assayed at 48 h. Error bars represent the s.e.m.

© Cancer Research Campaign 1997

**Figure 4** Apoptosis induced in PC-3M cells. Lane designations are: 123-bp DNA ladder standard (lane 1); controls (no drug, lane 2); 100 nM paclitaxel alone (lane 3); 30 μM quinacrine alone (lane 4); and 10 μM quinacrine with 10 nM paclitaxel (lane 5). DNA was loaded on a 1% agarose gel containing ethidium bromide, subjected to electrophoresis and photographed with a polaroid camera

**Figure 5** The effect of quinacrine and paclitaxel on the growth of DU145 xenografts in nude mice. Treatment groups (and their respective doses) are: controls (●, sterile water); quinacrine alone (■, 100 mg kg⁻¹), paclitaxel alone (▲, 1 mg kg⁻¹), quinacrine (50 mg kg⁻¹) + paclitaxel (0.5 mg kg⁻¹) concurrently (▼) and quinacrine (100 mg kg⁻¹) + paclitaxel (1 mg kg⁻¹) sequentially on alternate days (◆). Time (days) is plotted along the abscissa, and tumour size (mm³) along the ordinate. Treatment began after mice were randomized to assigned groups 5 days after cells were implanted subcutaneously in both flanks. Error bars indicate the s.e.m. ($n$ = 7–9 mice for each group). The arrow represents the start of treatment

### Synergy assessed by Chou analysis

By Chou analysis, most combination dose ratios of Q and P produced marked synergy, under both mutually exclusive and mutually non-exclusive conditions (data not shown), although apparent antagonism also occurred. Increasing doses of both drugs, in general, produces more growth inhibition (higher $F_a$), but synergy becomes more apparent at higher Q doses (5–10 μM), where the combination index was less than one for all dose combinations tested. Figure 2 plots the combination index (CI) values for a variety of Q/P ratios, grouped by varying background Q levels. In general, the higher Q levels (5 and 10 μM) are associated with more consistent synergism across the range of P levels tested, although synergism is seen with Q concentrations as low as 0.1 μM. At the lower Q doses (0.1, 0.5 and 1 μM), lower P doses are associated with antagonism. Taken together, these results suggest that a minimum threshold Q concentration of between 0.1 and 0.5 μM is required for synergy, and at this range P concentrations between 5 and 50 nM appear to be associated with the most synergy.

### Effect of sequence of drug administration on cell viability

Figure 3 depicts the effect on cell viability of changing the sequence of drug administration from 24 h Q followed by 24 h P, and the reverse. At 48 h, there is no effect of the sequence of administration of drugs on cell viability for PC-3M or DU145. The degree of growth inhibition of PC-3 cells increases when P is given before Q.

### Synergy of quinacrine- and paclitaxel-induced apoptosis

Figure 4 demonstrates apoptosis of PC-3M cells induced by 30 μM Q alone, 100 nM P alone and the combination of 10 μM Q with 10 nM P. Despite a markedly reduced dose of both drugs in combination, DNA fragmentation is still visible, indicating that the combination may be synergistic in inducing apoptosis. This observation was confirmed by an ELISA method, in which 2.5 μM Q combined with 25 nM P produced as much apoptosis as 5 μM Q alone and more apoptosis than 50 nM P alone (data not shown). In other experiments, the addition of 5 μM arachidonic acid was associated with a 37% reduction in apoptosis induced by the combination (data not shown), suggesting that the combination of Q and P does indeed perturb elements of the $PLA_2$ signal transduction pathway, the effects of which are partially reversed by the addition of exogenous arachidonic acid.

### Effect of lovastatin and quinacrine on PC-3

To confirm the hypothesis that quinacrine can enhance the action of inhibitors of *ras* protein function, we also used lovastatin, a competitive inhibitor of HMGCoA reductase and an inhibitor of *ras* signalling (Hohl and Lewis, 1995), concurrently with Q against PC-3 cells, and assessed the combination for synergy with Chou analysis. In general, the results suggest that this combination also has synergistic effects (data not shown). For lovastatin concentrations of 1 μM, all concentrations of Q (0.1–10 μM) were synergistic in inhibiting growth of PC-3 cells, while for lovastatin concentrations lower or higher than 1 μM, some ratios suggested mild synergy and others suggested mild antagonism (data not shown).

© Cancer Research Campaign 1997

**Table 2** Combination effects of quinacrine (Q) and paclitaxel (P) in various non-constant concentration ratios against PC-3, PC-3M and DU145

| Q (μM) | P (nM) | Combination index | | |
|---|---|---|---|---|
| | | PC-3 | PC-3M | DU145 |
| 0.1 | 0.5 | 6.37 ± 5.85 | 19.6 ± 18.7 | 0.22 ± 0.06 |
| 0.1 | 1 | 51.2 ± 50.3 | 38.8 ± 37.4 | 0.31 ± 0.12 |
| 0.1 | 5 | 6.35 ± 6.06 | 6.52 ± 3.23 | 0.84 ± 0.26 |
| 0.1 | 10 | 0.99 ± 0.64 | 1.20 ± 0.04 | 0.37 ± 0.04 |
| 0.1 | 50 | 1.93 ± 0.88 | 1.83 ± 0.21 | 1.25 ± 0.12 |
| 0.5 | 0.5 | 24.5 ± 18.3 | 20.8 ± 18.7 | 0.61 ± 0.07 |
| 0.5 | 1 | 2.94 ± 0.87 | 2.54 ± 0.97 | 0.82 ± 0.25 |
| 0.5 | 5 | 0.56 ± 0.24 | 0.36 ± 0.06 | 1.33 ± 0.36 |
| 0.5 | 10 | 0.53 ± 0.18 | 0.29 ± 0.03 | 0.41 ± 0.04 |
| 0.5 | 50 | 1.51 ± 0.47 | 0.79 ± 0.19 | 1.31 ± 0.17 |
| 1 | 0.5 | 1.57 ± 0.55 | 1.58 ± 0.71 | 3.17 ± 1.12 |
| 1 | 1 | 1.14 ± 0.44 | 1.07 ± 0.20 | 2.73 ± 1.37 |
| 1 | 5 | 0.46 ± 0.10 | 0.71 ± 0.25 | 1.74 ± 0.45 |
| 1 | 10 | 0.43 ± 0.07 | 0.51 ± 0.04 | 0.56 ± 0.09 |
| 1 | 50 | 1.04 ± 0.15 | 1.27 ± 0.17 | 1.47 ± 0.17 |
| 5 | 0.5 | 0.77 ± 0.10 | 0.60 ± 0.17 | 0.73 ± 0.07 |
| 5 | 1 | 0.81 ± 0.11 | 0.60 ± 0.16 | 0.80 ± 0.05 |
| 5 | 5 | 0.77 ± 0.09 | 0.70 ± 0.15 | 0.93 ± 0.11 |
| 5 | 10 | 0.84 ± 0.01 | 0.80 ± 0.08 | 0.68 ± 0.05 |
| 5 | 50 | 1.32 ± 0.15 | 1.72 ± 0.17 | 1.39 ± 0.17 |
| 10 | 0.5 | 0.39 ± 0.15 | 0.46 ± 0.08 | 0.51 ± 0.03 |
| 10 | 1 | 0.38 ± 0.14 | 0.50 ± 0.13 | 0.52 ± 0.02 |
| 10 | 5 | 0.41 ± 0.15 | 0.45 ± 0.10 | 0.64 ± 0.06 |
| 10 | 10 | 0.41 ± 0.17 | 0.45 ± 0.10 | 0.55 ± 0.02 |
| 10 | 50 | 0.61 ± 0.22 | 0.61 ± 0.09 | 0.99 ± 0.06 |

Combination index (CI) >1, = 1 and <1 indicate synergism, additive effect and antagonism respectively. CI values are reported ± s.e.m.

### Activity of paclitaxel and quinacrine against xenografts

Using RMANOVA, a statistically significant difference ($P < 0.05$, $q = 5.06$, Student–Newman–Keul's method) was found between the QP combination and the P alone group, suggesting that the combination was truly synergistic in vivo (Figure 5). In PC-3 and PC-3M xenografted mice, no differences between any of the treatment arms were seen (data not shown).

### Toxicity of paclitaxel and quinacrine

Other than a yellow pigmentation associated with Q treatment, noticeable after about 3 days, no obvious toxicity resulted from the combination of Q and P. For each group, the mean weight loss was less than 5% over the entire treatment period.

### DISCUSSION

We have shown that quinacrine and paclitaxel appear to act synergistically in inhibiting the in vitro growth of three hormone-independent human prostate cancer cell lines, PC-3, PC-3M and DU145. Our initial observations suggested that this synergy was most consistent at higher quinacrine concentrations (5–10 μM), raising the possibility that a threshold concentration of quinacrine is required for full expression of synergy. As $PLA_2$ has a role in many signal transduction pathways, we speculate that exceeding such a threshold may be required for suppression of its functions. Synergy was seen at quinacrine concentrations as low as 0.1 μM, indicating that the exact combination ratio of these drugs may not be an important determinant of their synergistic activity. Synergy was also seen in DU145-bearing mice, where the combination inhibited the growth of DU145 tumour size more effectively than either quinacrine or paclitaxel alone (Figure 5), the differences being statistically significant by RMANOVA (Heitjan et al, 1993), indicating true synergy. This statistical method takes into account the change in tumour size with time, similar to the way that all survival time for patients until the point of censorship is included in survival analyses in human trials. Although at half the dose intensity of each drug given alone, sequential treatment also appeared to inhibit tumour growth better than either drug alone, supporting the idea of synergy for the combination.

Synergistic activity was seen in DU145-bearing mice, despite our inability to keep to schedule owing to the development of venous thromboses in the tail veins from paclitaxel treatment. We therefore reasoned that synergy should still occur when the frequency of paclitaxel treatment was reduced. We chose PC-3 and PC-3M to test whether different scheduling would affect synergy, because we wanted to screen the combination against different cell lines and because the in vitro studies had suggested that the combination of quinacrine and paclitaxel was synergistic to a similar degree in each cell line, indicating that the actual cell line was unlikely to be important. However, we were surprised to find that no growth-inhibitory effect for any of the treatment arms was seen in PC-3- or PC-3M-bearing mice. The reasons for this are not clear, in view of clear synergy demonstrated for these cell lines in vitro. We speculate that treatment schedule may play an important role in the demonstration of synergy in vivo between quinacrine and paclitaxel, but are unable to make this conclusion because different cell lines were used.

Chou analyses can be difficult to interpret. In particular, the high CIs seen with the combination of either 0.1 μM or 0.5 μM quinacrine and 0.1 nM or 0.5 nM paclitaxel (Table 2) suggest marked antagonism, but these were also accompanied by large standard errors, suggesting that these results could have occurred by chance. From Table 2, CIs > 1 (indicating antagonism) tend to occur at ineffective concentrations of both drugs when used as single agents. This may result from the initial need to demonstrate sigmoid dose–response curves for each drug alone in a Chou synergy analysis, so the effect of low or ineffective drug doses may have a disproportionate impact on the conclusion. Consequently, small variations at the flat parts of the sigmoid dose–response curve, normally accepted as part of the limitations of the experimental procedure, can cause exaggerated interpretations of antagonism. Other investigators (Chou et al, 1993) have avoided this problem by choosing a fixed ratio of drug concentrations for testing synergy. However, this approach may have less clinical relevance because drug concentrations change with time in vivo. It is for this reason that we chose to test for synergy with non-constant ratios of both drugs.

The relative dose and effect of drugs used in synergy experiments are critical. Synergy can be shown mathematically with the Chou equation (Chou, 1991; Chou and Chou, 1987), if drug doses in combination are 50% of doses of each drug alone, provided the effect of the combination is equal to or better than for each drug alone (see Methods). In the apoptosis experiments, although the degree of apoptosis induced by the combination looked qualitatively less than for either quinacrine or paclitaxel alone (Figure 4), much less than 50% of the dose of each agent was used. Quantitating the apoptosis by spectophotometric means would not have been helpful, because both the drug doses and, as it happened, the degree of apoptosis, were different. By the ELISA

© Cancer Research Campaign 1997

method, however, we were able to show quantitatively that there was at least additive activity for the combination in inducing apoptosis. The finding that arachidonic acid can partially reverse the apoptosis induced by the combination of quinacrine and paclitaxel suggests involvement of the $PLA_2$ pathway. However, it would be simplistic to suggest that this was also evidence for the mechanism of synergy between quinacrine and paclitaxel, because both drugs have other documented actions. Further, *ras* also has other functions than progressing to $PLA_2$ activation (see Khosravi-Far and Der, 1994 for review), and $PLA_2$ itself is activated by other pathways (see Chang et al, 1987 for review). Nevertheless, the observation that lovastatin, another inhibitor of *ras* isoprenylation and function (Hohl and Lewis, 1995; Ruch et al, 1993), has synergistic activity in combination with quinacrine suggests that the *ras*/$PLA_2$ pathway might be a fruitful target for anti-cancer therapy.

Quinacrine enhances the action of paclitaxel, but the exact mechanism of action is unclear and may result from reasons other than its effect on the *ras* signalling pathway. Ford et al (1989) showed that quinacrine was able to reverse some of the effects of the mdr phenotype in doxorubicin-resistant MCF-7/DOX cells. However, mdr1 mRNA levels in primary prostate cancer, while common, are expressed at low levels as determined by RNA-polymerase chain reaction (PCR) (Siegsmund et al, 1994). Consequently, reversal of mdr phenotype effects by quinacrine may not play a major role in our results. Others (Zidovetzki et al, 1990) have found that quinacrine can alter membrane phospholipid function. This may be advantageous in that the non-specific nature of the disruption could also theoretically cause perturbation of *ras*, as well as $PLA_2$, function, both of which require relocation to the plasma membrane in order to be activated.

The prevalence of *ras* mutations in prostate cancer is of the order of 4% in American men and 25% in Japanese men (Isaacs et al, 1994). However, as these estimates were based on a spectrum of disease, from latent carcinoma to metastatic disease, the true incidence in HRPC is unknown. Because *ras* has been implicated in the progression to hormone-resistant disease (Voeller et al, 1991), and transfection of dominant-negative H-*ras* mutants into PC-3 cells has been shown to be very effective in inhibiting growth (Ogiso et al, 1994), further investigation on the role of *ras* in the development and growth of metastatic prostate cancer is warranted.

While a number of *ras* pathway inhibitors are presently in early clinical trials, a certain amount of clinical experience with paclitaxel and quinacrine already exists, and it seems reasonable to use these drugs for initial clinical trials to test the concept of combining *ras*/$PLA_2$ pathway inhibitors. The most clinically useful steady-state concentrations of quinacrine and paclitaxel associated with synergy may be around 0.5 μM and 5–10 nM respectively. As short infusions of paclitaxel at typical clinical doses (> 100 mg $m^{-2}$) are associated with much higher concentrations (Jamis-Dow et al, 1993) than represented by our in vitro studies, the conditions for synergy appear easily achievable. Further, because synergy was seen over a range of drug concentration ratios, faithful duplication of the in vitro conditions for synergy may not be required. Peak plasma concentrations of up to 140 ng $ml^{-1}$ (about 0.32 μM) for quinacrine are possible on a standard malaria regimen (Shannon et al, 1944), suggesting that a convenient design of a clinical trial for the combination of quinacrine and paclitaxel might employ chronic oral dosing for quinacrine together with standard doses and scheduling of paclitaxel. The significance of such a combination, if it proves to have activity in the clinical setting, lies in the possible improvement in toxicity profile over such combinations as estramustine and paclitaxel for prostate cancer (Hudes et al, 1995), as well as its potential application in the treatment of other *ras*-associated tumours, such as pancreatic and thyroid cancer.

### ACKNOWLEDGEMENTS

MC was the recipient of a 1995 HHMI programme summer research scholarship while at the University of Virginia. Part of this work was presented in abstract form at ASCO in Philadelphia, May, 1996.

### REFERENCES

Babb RR (1995) Giardiasis. Taming this pervasive parasitic infection. *Postgrad Med* **98**: 155–158

Chang J, Musser JH and McGregor H (1987) Phospholipase $A_2$: function and pharmacological regulation. *Biochem Pharmacol* **36**: 2429–2436

Chou JH (1991) Quantitation of synergism and antagonism of two or more drugs by computerized analysis. In *Synergism and Antagonism in Chemotherapy*, Chou TC and Rideout DC (eds), pp. 223–241. Academic Press: San Diego

Chou TC and Talalay P (1977) A simple generalized equation for the analysis of multiple inhibitions of Michaelis–Menten kinetic systems. *J Biol Chem* **252**: 6438–6442

Chou TC and Talalay P (1984) Quantitative analysis of dose–effect relationships: the combined effects of multiple drugs or enzyme inhibitors. *Adv Enzyme Regul* **22**: 27–55

Chou J and Chou TC (1987) *Dose Effect with Microcomputers, Manual and Software for IBM–PC*. Biosoft: Cambridge, UK

Chou TC, Tan QH and Sirotnak FM (1993) Quantitation of the synergistic interaction of edatrexate and cisplatin in vitro. *Cancer Chemother Pharmacol* **31**: 259–264

Chou TC, Motzer RJ, Tong Y and Bosl GJ (1994) Computerized quantitation of synergism and antagonism of paclitaxel, topotecan, and cisplatin against human teratocarcinoma cell growth: a rational approach to clinical protocol design. *J Natl Cancer Inst* **86**: 1517–1524

Danesi R, Figg DW, Reed E and Myers CE (1995) Paclitaxel (Taxol®) inhibits isoprenylation and induces apoptosis in PC-3 human prostate cancer cells. *Mol Pharmacol* **47**: 1106–1111

Eiseman JL, Eddington ND, Leslie J, Macauley C, Sentz DL, Zuhowski M, Kujawa JM, Young D and Egorin M (1994) Plasma pharmacokinetics and tissue distribution of paclitaxel in $CD_2F_1$ mice. *Cancer Chemother Pharmacol* **34**: 465–471

Fenton RG, Kung HF, Longo DL and Smith MR (1992) Regulation of intracellular actin polymerization by prenylated cellular proteins. *J Cell Biol* **117**: 347–356

Ford JM, Prozialeck WC and Hait WN (1989) Structural features determining activity of phenothiazines and related drugs for inhibition of cell growth and reversal of multidrug resistance. *Mol Pharmacol* **35**: 105–115

Hanada K, Kinoshita E, Itoh M, Hirata M, Kajiyama G and Sugiyama M (1995) Human pancreatic phospholipase $A_2$ stimulates the growth of human pancreatic cancer cell line. *FEBS Lett* **373**: 85–87

Hohl RJ and Lewis K (1995) Differential effects of monoterpenes and lovastatin on RAS processing. *J Biol Chem* **270**: 17508–17512

Heitjan DF, Manni A and Santen RJ (1993) Statistical analysis of *in vivo* tumor growth experiments. *Cancer Res* **53**: 6042–6050

Hudes GR, Nathan FE, Khater C, Greenberg R, Gomella L, Stern C and McAleer C (1995) Paclitaxel plus estramustine in metastatic hormone-refractory prostate cancer. *Semin Oncol* **22** (suppl. 12): 41–45

Isaacs WB, Morton RA, Bussemakers MJG, Brooks JD and Ewing CM (1994) Molecular biology of prostate cancer. *Semin Oncol* **21**: 514–521

Jamis-Dow CA, Klecker RW, Sarosy G, Reed E and Collins JM (1993) Steady-state plasma concentrations and effects of taxol for a 250 mg/$m^2$ dose in combination with granulocyte-colony stimulating factor in patients with ovarian cancer. *Cancer Chemother Pharmacol* **33**: 48–52

Khosravi-Far R and Der C (1994) The Ras signal transduction pathway. *Cancer Metast Rev* **13**: 67–89

Lin LL, Wartmann M, Lin AY, Knopf JL, Seth A and Davis RD (1992) $cPLA_2$ is phosphorylated and activated by MAP kinase. *Cell* **72**: 269–278

Murata K, Egami H, Kiyohara W, Oshima S, Kurizaki T and Ogawa M (1993) Expression of group II phosholipase $A_2$ in malignant and non-malignant human gastric mucosa. *Br J Cancer* **68**: 103–111

Ogiso Y, Sakai N, Watari H, Yokoyama T and Kuzumaki N (1994) Suppression of various human tumor cell lines by a dominant negative H-ras mutant. *Gene Therapy* **1**: 403–407

Pretlow TG, Delmoro CM, Dilley GG, Spadafora CG and Pretlow TP (1991) Transplantation of human prostatic carcinoma into nude mice in matrigel. *Cancer Res* **51**: 3814–3817

Ruch RJ, Madhukar BV, Trosko JE and Klaunig JE (1993) Reversal of *ras*-induced inhibition of gap–junctional intercellular communication, transformation, and tumorigenesis by lovastatin. *Mol Carcinogenesis* **7**: 50–59

Schiff P, Fant J and Horwitz S (1979) Promotion of microtubule assembly in vitro by Taxol. *Nature* **277**: 665–667

Schiff P and Horwitz SB (1980) Taxol stabilizes microtubules in mouse fibroblast cells. *Proc Natl Acad Sci USA* **77**: 1561–1565

Seigsmund MJ, Cardarelli C, Aksentijevich I, Sugimoto Y, Pastan I and Gottesman MM (1994) Ketoconazole effectively reverses multidrug resistance in highly resistant KB cells. *J Urol* **151**: 485–491

Shannon JA, Earle DP, Brodie BB, Taggart JV and Berliner RW (1944) The pharmacological basis for the rational use of atabrine in the treatment of malaria. *J Pharmacol Exp Ther* **81**: 307–330

Tokumoto H, Croxtall JD, Choudoury Q and Flower RJ (1993) Phospholipase $A_2$-induced stimulation of A549 lung adenocarcinoma cell line proliferation. *Biochim Biophys Acta* **1164**: 236–242

Voeller HJ, Wilding G and Gelman EP (1991) v-$ras^H$ expression confers hormone-independent in vitro growth to LNCaP prostate carcinoma cells. *Mol Endocrinol* **5**: 209–216

Wallace DJ (1994) Antimalarial agents and lupus. *Rheum Dis Clin N Am* **20**: 243–263

Yamashita SI, Yamashita JI and Ogawa M (1994) Overexpression of group II phospholipase $A_2$ in human breast cancer tissues is closely associated with their malignant potency. *Br J Cancer* **69**: 1166–1170

Zidovetzki R, Sherman IW, Maguire PA and De Boeck H (1990) A nuclear magnetic resonance study of the interactions of the antimalarials chloroquine, quinacrine and mefloquine with lipids extracted from normal human erythrocytes. *Mol Biochem Parasitol* **38**: 33–40

*© Cancer Research Campaign 1997*